# Co-occurrence of Point Mutations in the Voltage-Gated Sodium Channel of Pyrethroid-Resistant *Aedes aegypti* Populations in Myanmar

**DOI:** 10.1371/journal.pntd.0003032

**Published:** 2014-07-31

**Authors:** Hitoshi Kawada, Sai Zaw Min Oo, Sein Thaung, Emiko Kawashima, Yan Naung Maung Maung, Hlaing Myat Thu, Kyaw Zin Thant, Noboru Minakawa

**Affiliations:** 1 Department of Vector Ecology and Environment, Institute of Tropical Medicine, Nagasaki University, Nagasaki, Japan; 2 Medical Entomology Research Division, Department of Medical Research (Lower Myanmar), Yangon, Myanmar; 3 The Global Center of Excellence Program, Nagasaki University, Nagasaki, Japan; Mahidol University, Thailand

## Abstract

**Background:**

Single amino acid substitutions in the voltage-gated sodium channel associated with pyrethroid resistance constitute one of the main causative factors of knockdown resistance in insects. The *kdr* gene has been observed in several mosquito species; however, point mutations in the para gene of *Aedes aegypti* populations in Myanmar have not been fully characterized. The aim of the present study was to determine the types and frequencies of mutations in the para gene of *Aedes aegypti* collected from used tires in Yangon City, Myanmar.

**Methodology/Principal Findings:**

We determined high pyrethroid resistance in *Aedes aegypti* larvae at all collection sites in Yangon City, by using a simplified knockdown bioassay. We showed that V1016G and S989P mutations were widely distributed, with high frequencies (84.4% and 78.8%, respectively). By contrast, we were unable to detect I1011M (or I1011V) or L1014F mutations. F1534C mutations were also widely distributed, but with a lower frequency than the V1016G mutation (21.2%). High percentage of co-occurrence of the homozygous V1016G/S989P mutations was detected (65.7%). Additionally, co-occurrence of homozygous V1016G/F1534C mutations (2.9%) and homozygous V1016G/F1534C/S989P mutations (0.98%) were detected in the present study.

**Conclusions/Significance:**

Pyrethroid insecticides were first used for malaria control in 1992, and have since been constantly used in Myanmar. This intensive use may explain the strong selection pressure toward *Aedes aegypti*, because this mosquito is generally a domestic and endophagic species with a preference for indoor breeding. Extensive use of DDT for malaria control before the use of this chemical was banned may also explain the development of pyrethroid resistance in *Aedes aegypti*.

## Author Summary

The use of pyrethroids with high killing activity has accelerated the development of pyrethroid resistance in vector mosquitoes. The knockdown resistance (*kdr*) allele contains a single amino acid substitution in the voltage-gated sodium channel and is one of the main causative factors of pyrethroid resistance in insects. We investigated the distribution of the *kdr* gene in *Aedes aegypti* larvae collected from used tires in Yangon City, Myanmar. We detected three patterns of co-occurrence of point mutations, namely, V1016G/S989P with wide distribution, and small number of V1016G/F1534C and V1016G/F1534C/S989P, both of which were first found in the field collected *Ae. aegypti* population. The use of pyrethroids and/or DDT for malaria control is thought to be one of the main causes of pyrethroid resistance in *Aedes aegypti*. Insecticide treatment for malaria vector control seems to have been intensively conducted in the interior and along the periphery of human habitation areas, where the breeding and resting sites of *Aedes aegypti* are located.

## Introduction

Pyrethroids constitute a group of chemicals that are structurally modified by natural pyrethrum, and are composed of two main types. Most of the 1st generation pyrethroids are knockdown agents, which possess high knockdown activity but low killing activity; the second generation pyrethroids possess high killing activity. The use of knockdown agents such as *d*-allethrin and metofluthrin—which generally exhibit low stability in the environment—as “spatial repellents” is biorational, because it causes low selection pressure on insect populations, thereby minimizing the development of physiological resistance. On the other hand, killing agents such as permethrin, deltamethrin, cypermethrin, and λ-cyhalothrin generally exhibit high photostability, and this enables their outdoor use in agriculture and their predominant application as vector control agents. Globally, photostable pyrethroids comprise 40% of the insecticides used annually for indoor residual spraying against malaria vectors [1]. However, the high stability and killing efficacy of pyrethroids has accelerated the development of pyrethroid resistance in vector mosquitoes [2].

Resistance to photostable pyrethroids is a major problem for the vector control program. Pyrethroid resistance involves two main mechanisms, namely, enhanced metabolic detoxification and insensitivity of target sites. Single amino acid substitutions in the voltage-gated sodium channel, known as *kdr* mutations, constitute one of the main causative factors of pyrethroid resistance. This *kdr*-type resistance has been observed in several mosquitoes, including *Anopheles gambiae* Giles [3], *Anopheles stephensi* Liston [4], *Culex quinquefasciatus* Say [5], and *Aedes aegypti* (L.) [6]. Several mutations in segment 6 of domain II of the voltage-gated sodium channel were reported to play important roles in pyrethroid resistance of *Aedes aegypti* (I1011M, I1011V, V1016G, and V1016I) [6]–[8]. Recently, Yanola et al. identified a novel F1534C mutation in segment 6 of domain III in DDT/permethrin-resistant *Aedes aegypti* [9], [10]. Harris et al. reported that the F1534C mutation was strongly correlated with resistance to DDT and pyrethroid [11]. The S989P mutation in domain II of the voltage-gated sodium channel gene, which occurs in deltamethrin-resistant *Aedes aegypti*, is another principal *kdr* mutation that regulates pyrethroid resistance in mosquitoes [12]. Yanola et al. [10] reported the existence of the F1534C mutation in *Aedes aegypti* from Yangon City, Myanmar. However, to the best of our knowledge, no other detailed genetic analyses of point mutations in the voltage-gated sodium channel of *Aedes aegypti* colonies in Myanmar have been performed.

The objective of the present study was to elucidate the mechanisms of pyrethroid resistance in *Aedes aegypti* collected from used tires in Yangon City, Myanmar, by analyzing the presence of mutations in the voltage-gated sodium channel gene. Our targets were the four most frequent amino acid replacements in S989, I1011, L1014, and V1016—all of which are located in the area of segment 6 of domain II—and also a recently identified amino acid replacement at F1534—located in the area of segment 6 of domain III.

## Methods

### Collection of *Aedes aegypti* larvae from used tires and application of a simplified knockdown bioassay

We drove along the main roads in five urban townships (Tharketa, Thingangyun, North Dagon, South Okklapa, and Tarmwe) and two suburban townships (Thanlyin and Hlegu) in Yangon City, from September 26 to October 4, 2013, and collected mosquito larvae from used tires. Whenever we encountered used tires, most of which were found along the periphery of repair shops, we recorded the geographical location of the collection site by using a global positioning system (GPS). In addition, we took photographs of the surrounding area to record environmental characteristics such as vegetation, urbanization, etc. We collected mosquito larvae from the tires with netting. We performed a simplified bioassay for the detection of knockdown susceptibility in the *Aedes* larvae on the day of collection, according to Kawada et al. [13]. Each larva was placed in a glass vial containing 20 mL of water. An emulsifiable concentrate of 90% *d*-T_80_-allethrin (Pynamin Forte 90EC, lot. 7304M901, Sumitomo Chemical Co., Ltd., Tokyo, Japan) was diluted with water to obtain a 250 ppm solution. After releasing the larva, we added 32 µL or 8 µL of the solution to each vial, to obtain a concentration of 0.4 ppm or 0.1 ppm, respectively. We used 10 larvae for each concentration regime, and observed larval knockdown for 30 min. Larvae that sank to the bottom of the glass vial and could not swim, float, or were paralyzed were judged as knocked down larva; we recorded the time to knockdown for each of these larva. We scored the median knockdown time (KT_50_), i.e., the time required for 50% knockdown of larvae used for each concentration regime, according to the six following categories: 1, <5 min; 2, 5 to <10 min; 3, 10 to <15 min; 4, 15 to <20 min; 5, 20 to <30 min; and 6, ≥30 min. We calculated the susceptibility index as the product of the scores at 0.1 ppm and 0.4 ppm. Thus, mosquitoes with a susceptibility index of 1 were considered to be the most susceptible to *d*-allethrin, and those with a susceptibility index of 36 were considered to be the least susceptible to *d*-allethrin. After the bioassay, we identified each larva according to the keys provided by Rattanarithikul et al. [14]. The larvae were then stored in a 1.5-mL plastic vial containing ethanol solution, for subsequent polymerase chain reaction (PCR) analysis.

### Analysis of the frequency of point mutations

To test for the presence of point mutations at S989, I1011, L1014, V1016, and F1534 in *Aedes aegypti*, we used PCR and direct DNA sequencing. We performed the sequencing on 20 larvae/collection site, which were randomly selected after the bioassay. The mosquito sample (i.e., a larva immersed in ethanol) was lightly dried on a paper towel and placed in a 1.5-mL PCR reaction tube. The sample was homogenized in a mixed solution of extraction solution (40 µL) plus tissue-preparation solution (10 µL) (REDExtract-N-Amp Tissue PCR Kit; Sigma, St. Louis, MO) for extraction of DNA. The solution was heated at 95°C for 3 min and neutralized. Initial amplification was carried out using the primers AaSCF1 (AGACAATGTGGATCGCTTCC) and AaSCR4 (GGACGCAATCTGGCTTGTTA) for S989P, I1011M (or V), L1014F, and V1016G (or I); or AaSCF7 (GAGAACTCGCCGATGAACTT) and AaSCR7 (GACGACGAAATCGAACAGGT) for F1534C. The PCR mixture contained 4 µL of REDExtract-N-Amp ReadyMix (Sigma), 0.5 µM of each primer, and 1 µL of the DNA template in a total volume of 10 µL. PCR was performed under the following conditions: initial denaturation at 94°C for 3 min; 35 cycles each of 94°C for 15 s, 55°C for 30 s, and 72°C for 30 s; and a final elongation step at 72°C for 10 min. The amplified fragments of the expected size were purified with ExoSAP-IT (USB Corporation, Cleveland, OH) at 37°C for 30 min, and then 80°C for 15 min. DNA sequencing was carried out by using the primers AaSCF3 (GTGGAACTTCACCGACTTCA) and AaSCR6 (CGACTTGATCCAGTTGGAGA) for S989P, I1011M (or V), L1014F; and V1016G (or I), or AaSCR8 (TAGCTTTCAGCGGCTTCTTC) for F1534C. A BigDye Terminator v 3.1 Cycle Sequencing Kit (Applied Biosystems Japan Ltd., Tokyo, Japan) was used for DNA sequencing, according to the manufacturer's instructions. Two micromoles of each primer were added to a tube, to give a total mixture volume of 10 µL. PCR was performed under the following conditions: initial denaturation at 96°C for 1 min; 25 cycles each of 96°C for 10 s, 50°C for 5 s, and 60°C for 2 min. Direct DNA sequencing was performed on the 3730 DNA Analyzer (Applied Biosystems Japan Ltd.). The electropherogram of the targeted amino acid replacement was analyzed with MEGA 4.0 public domain software (http://www.megasoftware.net/). The unique DNA haplotype sequences were deposited in GenBank.

### Statistical analysis

The susceptibility index and the corresponding frequency of point mutations for each collection point were plotted on a shape file map (DIVA-GIS, http://www.diva-gis.org/gdata) by using ArcGIS 10.2 (ESRI Japan Corp., Tokyo, Japan). An independent chi-squared test was performed for the number of larvae possessing V1016G and F1534C, or V1016G and S989P.

## Results

**Figure 1:**
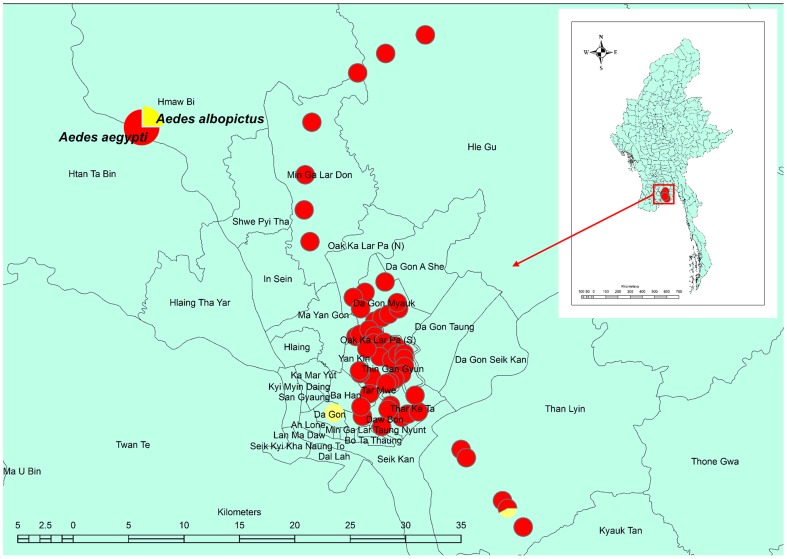
Distribution of *Aedes aegypti* and *Aedes albopictus* larvae. The larvae were collected from used tires in Yangon City. Each circle indicates the species composition of the two species.

**Figure 2:**
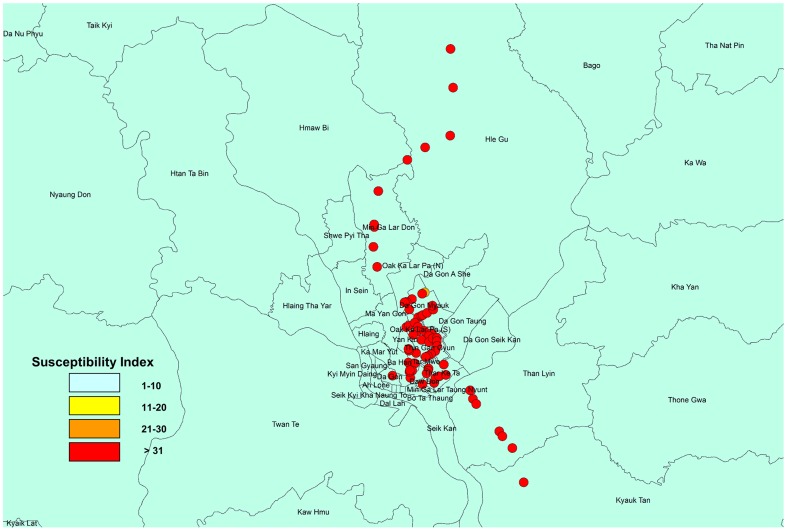
Distribution of susceptibility indices in *Aedes aegypti* larvae. The susceptibility index was determined by using the simplified knockdown bioassay. The color in each circle indicates the susceptibility index. The larger the index, the lower the susceptible to *d*-allethrin.

Figure 1 shows the distribution pattern of *Aedes aegypti* and *Aedes albopictus* Skuse larvae collected from used tires. We collected 2,400 larvae, among which 2,332 were *Aedes aegypti*, 29 were *Aedes albopictus*, and 39 were other species, including *Culex quinquefasciatus* Say. We identified *Aedes aegypti* larvae in 55 of the 57 collection sites, indicating the predominance of this mosquito; by contrast, we identified *Aedes albopictus* larvae in only three sites. Figure 2 shows the distribution of the susceptibility indices of *Aedes aegypti* larvae, as determined by the simplified knockdown bioassay. With the exception of a single location in North Dagon (susceptibility index, 30), the maximum susceptibility index (36) was recorded for *Aedes aegypti* larvae in 54 of the 55 sites where this species was collected. The percentages of knocked down larvae at 0.4 ppm of *d*-allethrin, determined 30 min after treatment, were 0% for 42 collection sites and 10–30% for the remaining 12 collection sites. The susceptibility index of *Aedes albopictus* collected at a single site in Dagon area was 18.

**Figure 3:**
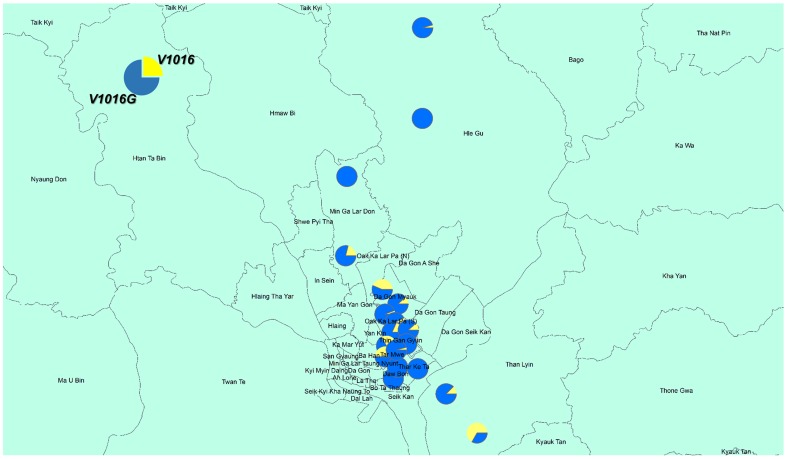
Distribution of the V1016G point mutation in *Aedes aegypti* larvae. The larvae were collected from used tires in Yangon City. Each circle indicates the allelic composition of the point mutations/wild types.

**Figure 4:**
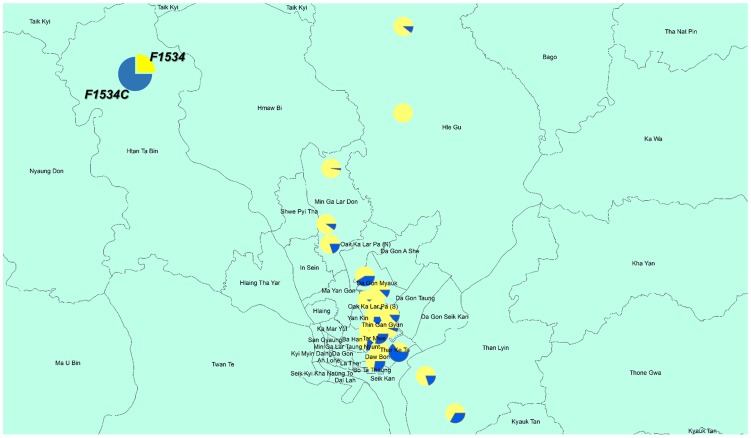
Distribution of the F1534C point mutation in *Aedes aegypti* larvae. The larvae were collected from used tires in Yangon City. Each circle indicates the allelic composition of the point mutations/wild types.

Figures 3 and 4 show the distribution of frequencies of point mutations at V1016, and F1534 in *Aedes aegypti* collected from used tires. We detected no mutations at I1011 and L1014 among 281 and 271 larvae sequenced, respectively. On the other hand, for V1016G, we detected 230 homozygous and 21 heterozygous mutations among 285 larvae sequenced (accession No. AB914689, AB914690). The frequency and % homozygous of the V1016G point mutation were 84.4% and 80.7%, respectively (Figure 3). For S989P, we detected 140 homozygous and 64 heterozygous point mutations among 218 larvae sequenced. The frequency and % homozygous of the S989P mutation were 78.8% and 64.2%, respectively. For the recently identified F1534C point mutation, we detected 16 homozygous and 105 heterozygous point mutations among 323 larvae sequenced (accession No. AB914687, AB914688). The frequency and % homozygous of the F1534 mutation were 21.2% and 5.0%, respectively (Figure 4). Although the number of sampling was few, no point mutation was detected in 20 larvae of *Ae. albopictus*.

**Figure 5:**
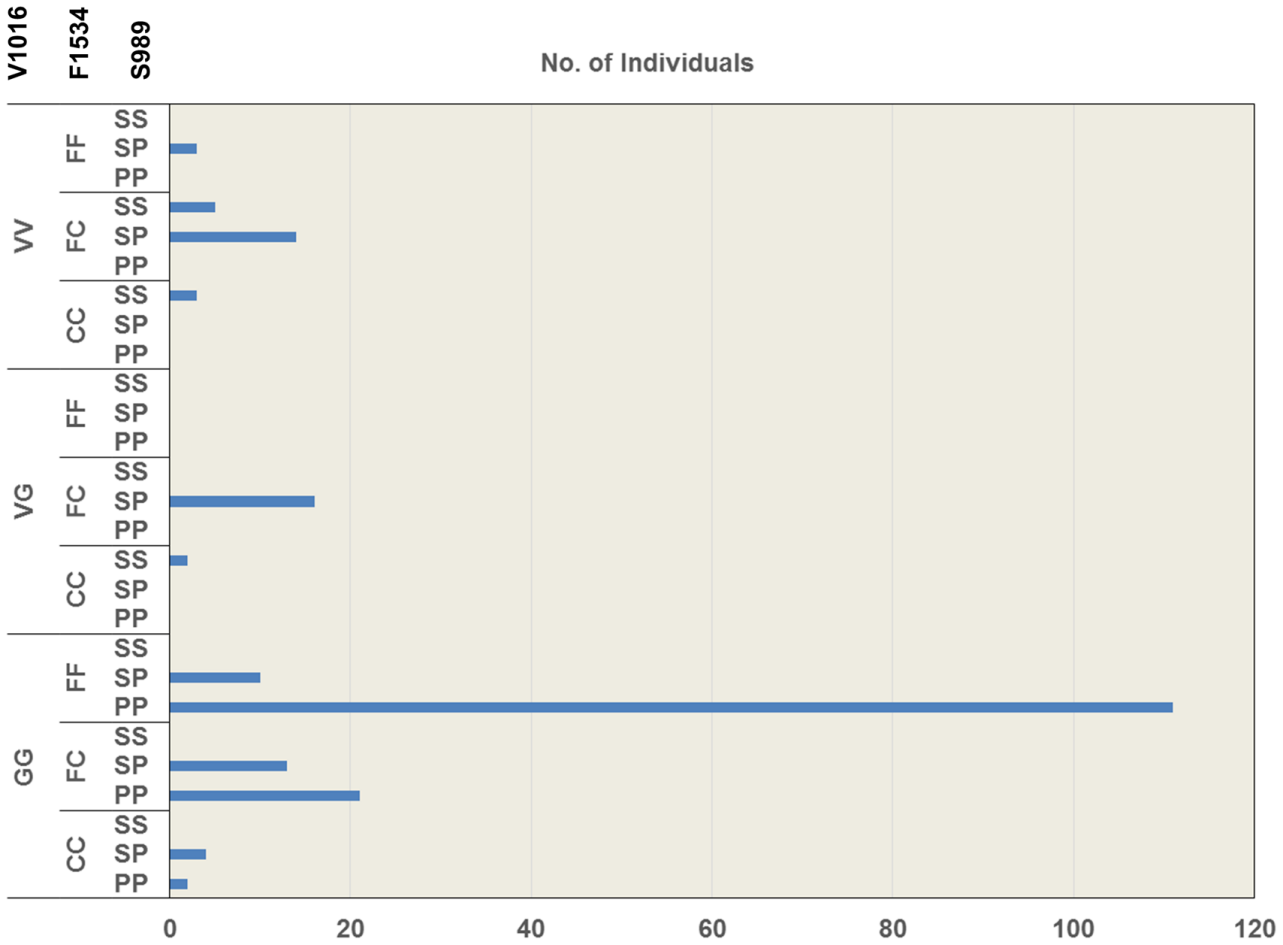
Genotyping of the V1016, F1534, and S989 point mutations in *Aedes aegypti* larvae. The larvae were collected from used tires in Yangon City. The total number of larval samples used for the analysis was 204.

We detected three patterns of co-occurrence of point mutations; V1016G/F1534C, V1016G/S989P, and V1016G/F1534C/S989P. We analyzed the haplotypes of each gene among 204 mosquitoes (Figure 5). Our results revealed that the percentage of larvae possessing the homozygous V1016G mutation and the wild type 1534F was significantly higher (59.3%) than the percentage of larvae possessing the homozygous V1016G and F1534C mutations (2.9%) (P<0.01, χ^2^ = 72.6). By contrast, the percentage of co-occurrence of the homozygous V1016G and S989P mutations was significantly high (65.7%) (P<0.01, χ^2^ = 135). Additionally, co-occurrences of the above three homozygous point mutations (V1016G/F1534C/S989P) were detected in 2 individuals among 204 individuals analyzed (0.98%).

## Discussion

In the present study, we detected high frequencies of the V1016G (84.4%) and S989P (78.8%) mutations in *Aedes aegypti* collected from used tires in Yangon City. Additionally, we detected a relatively low allelic frequency of the F1534C mutation (21.2%). The I1011 and L1014 point mutations were undetected in *Aedes aegypti* in the study area.

High frequencies, namely, 3.1–61.8% [15] and 23% [16], of the V1016G mutation in *Aedes aegypti* were reported in Thailand. The V1016G mutation was also found in Vietnam; however, the distribution was limited and the frequency of the point mutation was relatively low (2 heterozygotes were found among 10 larvae collected at a single site in Quang Ngai Province) [17]. At present, no other report of this point mutation in another Southeast Asian country has been published. The V1016I and I1011 (M or V) mutations seem to be widely distributed with high frequency in Latin American countries [7], [11], [18]–[21]. It is interesting to speculate why the V1016G point mutation occurs with high frequency in Myanmar and Thailand. Myanmar shares the longest border (2107 km) with Thailand among the neighboring countries, and therefore the two countries may share environmental, sociological, and biological similarities. Further studies to clarify the genetic relationships between two *Aedes aegypti* populations in Myanmar and Thailand, which may have had exchange or introgression of genes, are required.

After the first description of the newly identified F1534C point mutation in *Aedes aegypti* collected in Thailand [9], [10], the same mutation was reported in Vietnam [17]; Brazil; Venezuela; Madeira Island, Portugal [22]; and Grand Cayman Island, UK [11]. In the present study, we determined a moderate frequency of this point mutation in Yangon City (21.2%); this frequency was of a comparable level to that recorded in Vietnam (average, 21.6%) [17], but lower than the frequencies recorded in Thailand (59–79%) [10], Grand Cayman Island (68%) [11], and Venezuela (100%) [22]. Additionally, the same mutation was recently reported in another DHF vector, *Aedes albopictus*, at high frequency (73%) [23]. Elucidation of the worldwide distribution of the F1534C mutation in *Aedes aegypti* and *Aedes albopictus* will provide a valuable insight into DHF epidemiology, and yield useful information for vector control programs.

In the present study, we detected three patterns of co-occurrence of point mutations, namely, V1016G/S989P, V1016G/F1534C, and V1016G/F1534C/S989P. The S989P mutation has always been linked to the V1016G mutation; however, V1016G has sometimes been found in the absence of S989P [12]. The observed co-occurrence of the V1016G and S989P mutations with high frequency in Myanmar correspond to the former scenario. Srisawat et al. indicated that the co-occurrence of V1016G and S989P enhanced the resistance of *Aedes aegypti* to deltamethrin [12]. However, the authors were unable to determine the effect of the single S989P mutation on pyrethroid sensitivity, and were therefore unable to provide direct evidence of how the two mutations were associated with resistance. Recently, Du et al. reported that the co-occurrence of the V1016G and S989P mutations was not additive or synergistic to the V1016G mutation; however, the S989P mutation was concurrent with the V1016G mutation in a pyrethroid-resistant population of *Aedes aegypti* [24]. Further studies regarding the synergistic role of S989P on V1016G are required.

Interestingly, Stenhouse et al. [15] reported that, in an *Aedes aegypti* population in Thailand, wild type 1016V mosquitoes were homozygous for the F1534C mutation (43.5%); further, all 1016 heterozygous mosquitoes were 1534 heterozygous (38.8%), and homozygous V1016G mosquitoes were homozygous for the wild type 1534F (17.6%). These findings indicate that the wild type 1016V + homozygous F1534C, the homozygous V1016G, and the wild type 1534F exist with equal frequencies. On the contrary, in the present study, we detected dominance of the homozygous V1016G + homozygous wild type 1534F (59.3%); further, the percentage of mosquitoes possessing the wild type 1016V + homozygous F1534C mutations was low (1.5%). Additionally, we detected a small number of mosquitoes possessing the homozygous V1016G + F1534C (2.9%) and V1016G + F1534C + S989P (0.98%). Recently, Linss et al. [25] detected the co-occurrence of V1016I and F1534C point mutations in *Aedes aegypti* populations in Brazil, and reported that the widespread distribution of the wild type 1016V + homozygous F1534C and the homozygous V1016I + F1534C in the country during the previous decade. Interestingly, the frequency of mosquitoes with the homozygous V1016I + F1534C mutation in Brazil (average 20.5%) was higher than the reported frequencies of mosquitoes with the homozygous V1016G + F1534C. In Thailand, the F1534C mutation is considerably more common than the V1016G mutation [10], [15]. Mosquitoes with the homozygous F1534C mutation are thought to be susceptible to deltamethrin [15]. The I1011M and F1534C mutations seem to play a role in decreasing susceptibility to Type I pyrethroids, such as permethrin; on the other hand, the L1014F (or S) and V1016G mutation play important roles in the insensitivity of the voltage-gated sodium channel to Type II pyrethroids, such as deltamethrin [24], [26]. Du et al. [24] indicated that the contribution of the V1016I mutation to pyrethroid resistance was lower than that of the V1016G mutation. Therefore, in the *Aedes aegypti* population investigated in the present study, the higher frequency of occurrence of the V1016G mutation than the F1534C mutation may indicate a history of pyrethroid treatment as part of vector control programs in the study sites. First, the use of DDT or Type I pyrethroids might select F1534C mutations, then V1016G has been selected by the following use of type II pyrethroids. The possible increase of the homozygous V1016G and F1534C mutations associated with the S989P point mutation under increasing pyrethroid selection pressure may represent the worst case scenario for vector control programs.

The first outbreak of DHF was recorded in Myanmar in 1969. Since then, DHF has spread to 12 out of 14 states. During 1970–1991, 83381 cases, causing 3243 deaths were recorded [27]. The principal vector of DHF is *Aedes aegypti*, with *Aedes albopictus* acting as a secondary vector in suburban and rural areas. The growth of economic activities and traditional water storage practices increased the availability of vector breeding sites, thereby increasing the incidence of DHF cases from 5621 in 2005 to 11,049 in 2006 [28]. The *Aedes* mosquito control unit was established in 1968, and several feasibility studies aimed at the development of *Aedes aegypti* control methodology—including insecticide treatment, biological control by using larvivorous fish, environmental improvement, and health education—were undertaken [27]. However, information regarding insecticide usage for DHF vector control in Myanmar is lacking, and few studies concerning the insecticide resistance of DHF vectors have been published. Treatment of pyriproxyfen sand granules and BTI, and the use of these chemicals in combination with biological measures—such as the release of dragon fly nymphs [29]—seemed to be acceptable to the residents of Yangon City [30]. During 1988–2005, 510 t of organochlorine insecticides, 360 kg of organophosphate insecticides, and 8157 kg of pyrethroid insecticides were used for malaria control in Myanmar. By contrast, only 1.2 t of organophosphate insecticides were used for DFH control during the same period. These data indicate that large amounts of organochlorine insecticides, including DDT, were used in Myanmar until the use of these insecticides was banned in 2003 [1]. The ban on organochlorine insecticides was introduced later in Myanmar (2003) and Thailand (2001) than in other Southeast Asian countries. Thaung et al. reported that DDT was routinely used for the control of the rat flea, *Xenopsylla cheopsis* (Rothschild), and that periodical monitoring of insecticide susceptibility in Yangon City indicated DDT resistance in rat fleas. The authors further suggested the existence of resistance to DDT, and lack of resistance to other types of insecticides, in *Aedes aegypti* [31].

In the present study, we determined high pyrethroid resistance in the larvae of *Aedes aegypti*. Interestingly, we recorded the maximum susceptibility index (36) in almost all of the collection sites. We also noted high DDT and pyrethroid (permethrin and deltamethrin) resistance in adult *Aedes aegypti* collected in the seven townships surveyed (unpublished data). Vu et al. [32] and Huber et al. [33] concluded that prolonged use of pyrethroids for malaria control may be one of the causative factors of pyrethroid resistance in *Aedes aegypti* in Vietnam. Since the abandonment of DDT sprays in Vietnam in 1995, a large number of photostable pyrethroids (λ-cyhalothrin, α-cypermethrin, deltamethrin, and permethrin) have been used to treat the interiors of houses as a residual spray, and to manufacture pyrethroid-impregnated bed nets for malaria control [1], [34]–[36]. In Myanmar, pyrethroid insecticides were first used for malaria control in 1992, and have since been constantly used (>500 kg annually). Pyrethroid treatment for malaria vector control seems to have been intensively conducted in the interior and along the periphery of human habitation areas, where the breeding and resting sites of *Aedes aegypti* are located. This intensive usage may explain the strong selection pressure toward *Aedes aegypti*, because this mosquito is generally a domestic and endophagic species with a preference for indoor breeding [37]–[40]. Extensive use of DDT for malaria control before the use of this chemical was banned may also explain the development of pyrethroid resistance in *Aedes aegypti*, because the target site (i.e., the voltage-gated sodium channel) is common to DDT and pyrethroids.

Pyrethroids represent one of the most promising countermeasures for controlling malaria, DHF, and other arthropod-borne diseases. Currently, there are no suitable chemical substitutes for pyrethroids, and therefore pyrethroid resistance will be a major problem for vector control programs [41]. The development of new chemicals with novel modes of action, which can be substituted for conventional insecticides, is essential. Transitional life-prolonging measures for conventional 1st generation pyrethroids that belong to the photo-unstable knockdown agent groups and are used as “spatial repellents” [42], rotational use of plural insecticides with different mode of actions, and basic biochemical and genetic research to support the above strategies, are crucial to the effective management of insecticide resistance.
